# Serum albumin to globulin ratio is related to cognitive decline via reflection of homeostasis: a nested case-control study

**DOI:** 10.1186/s12883-016-0776-z

**Published:** 2016-12-08

**Authors:** Teruhide Koyama, Nagato Kuriyama, Etsuko Ozaki, Daisuke Matsui, Isao Watanabe, Fumitaro Miyatani, Masaki Kondo, Aiko Tamura, Takashi Kasai, Yoichi Ohshima, Tomokatsu Yoshida, Takahiko Tokuda, Ikuko Mizuta, Shigeto Mizuno, Kei Yamada, Kazuo Takeda, Sanae Matsumoto, Masanori Nakagawa, Toshiki Mizuno, Yoshiyuki Watanabe

**Affiliations:** 1 Department of Epidemiology for Community Health and Medicine, Kyoto Prefectural University of Medicine, 465 Kajii-cho, Kamigyo-ku, Kyoto, 602-8566 Japan; 2 Department of Dental Medicine, Kyoto Prefectural University of Medicine, 465 Kajii-cho, Kamigyo-ku, Kyoto, 602-8566 Japan; 3 Department of Neurology, Kyoto Prefectural University of Medicine, 465 Kajii-cho, Kamigyo-ku, Kyoto, 602-8566 Japan; 4 Department of Pharmacology, Kyoto Prefectural University of Medicine, 465 Kajii-cho, Kamigyo-ku, Kyoto, 602-8566 Japan; 5 Department of Molecular Pathobiology of Brain Diseases, Kyoto Prefectural University of Medicine, 465 Kajii-cho, Kamigyo-ku, Kyoto, 602-8566 Japan; 6 Endoscopy Department, Kindai University Nara Hospital, 1248-1 Otoda-cho, Ikoma, 630-0293 Japan; 7 Department of Radiology, Kyoto Prefectural University of Medicine, Kyoto, Japan; 8 Kyoto Industrial Health Association, 67 Nishinokyo Kitatsuboi-cho, Nakagyo-ku, Kyoto, 604-8472 Japan; 9 Director of North Medical Center, Kyoto Prefectural University of Medicine, 481 Aza-Otokoyama, Yosano-cho, Yosa-gun, Kyoto, 629-2261 Japan

**Keywords:** Albumin to globulin ratio, Cognitive decline, *Helicobacter pylori*, Homeostatic alteration, Mini-mental state examination

## Abstract

**Background:**

Recent research suggests that several pathogenetic factors, including aging, genetics, inflammation, dyslipidemia, diabetes, and infectious diseases, influence cognitive decline (CD) risk. However, no definitive candidate causes have been identified. The present study evaluated whether certain serum parameters predict CD.

**Methods:**

A total of 151 participants were assessed for CD using the Mini-Mental State Examination (MMSE), and 34 participants were identified as showing CD.

**Results:**

Among CD predictive risk factors, *Helicobacter pylori* seropositivity was significantly predictive of CD risk, more so than classical risk factors, including white matter lesions and arterial stiffness [adjusted odds ratio (OR) = 4.786, 95% confidence interval (CI) = 1.710–13.39]. A multivariate analysis indicated that the albumin to globulin (A/G) ratio was the only factor that significantly lowered CD risk (OR = 0.092, 95% CI = 0.010–0.887). A/G ratio also was positively correlated with MMSE scores and negatively correlated with disruption of homeostatic factors (i.e., non-high-density lipoprotein, hemoglobin A1c, and high-sensitive C-reactive protein).

**Conclusions:**

The current study results suggest that the A/G ratio is related to cognitive decline and may reflect homeostatic alterations.

## Background

Improvements in health care support have greatly extended average life expectancy, resulting in a substantial increase in the number of elderly individuals worldwide. Some forms of memory impairment are observed among elderly adults and can be predictive of age-related cognitive decline associated with Alzheimer’s disease (AD) [1] and other dementias. Rate of memory impairment varies based on several factors, including age, sex, types of cognitive tasks assessed, education, and emotional state [2]. Previous reports have noted several causes for cognitive decline (CD). For instance, infection can cause both direct and indirect decrements. The association between *Helicobacter pylori* (*H. pylori*) infection and AD has recently been addressed [3], and other infections [i.e., *Chlamydia pneumoniae* (*C. pneumoniae*), cytomegalovirus, and herpes simplex virus type1] may influence AD manifestation [4]. Furthermore, inflammation-mediated damage in the apolipoprotein E (ApoE) allele 4 suggests a plausible marker for cognitive impairment, possibly due to increased viral replication, which could eventually lead to AD [5]. One way to affect this relationship is by controlling risk factors (e.g., diabetes, cholesterol, hypertension, stroke, or smoking) that could help alleviate physiological dementia risk factors [6]. A common factor is chronic and systemic inflammation, which leads to increased levels of several proinflammatory cytokines that subsequently promote CD progression [7]. Chronic and systemic inflammation also induces atherosclerosis [8] and atherosclerosis-promoted cognitive impairment [9].

There is growing interest in identifying individuals who have not yet demonstrated CD but could be at greater risk for developing dementia. This is because cognitive impairment responds much better to treatment during early compared to advanced illness stages. With substantial increases in dementia incidence, early detection of possible precursors, diagnostics, treatment, and control of modifiable risk factors are highly important [10]. Insight is needed regarding the specific risk factors that predict CD incidence. Elucidation of these factors will help identify individuals with CD who are at the highest risk for developing AD in the near future.

Thus, the aim of the present nested case control study was to evaluate whether certain serum parameters, commonly measured during routine health checkups including magnetic resonance imaging (MRI) and pulse wave velocity as a marker of arterial stiffness, could be viable predictors of CD incidence.

## Methods

### Study participants

The present study consisted of self-administered questionnaires and medical examinations, including blood tests, conducted at the Kyoto Industrial Health Association. From 2003 to 2004, 488 Japanese participants completed a baseline epidemiological survey [11]. Basic cognitive functioning was assessed for 273 participants from 2006 to 2008 and for 290 participants from 2012 to 2014. A group of 151 participants (101 men and 50 women), with normal cognition in 2006–2008, attended follow-up visits during both the 2006–2008 and 2012–2014 periods. We included all of these 151 patients in our study in order to avoid selection bias. The Ethics Board from the Kyoto Prefectural University of Medicine approved the study protocol (G-144). After we explained the purpose of the study, written informed consent was obtained from all participants.

### Cognitive testing

The Mini-Mental State Examination (MMSE) is a brief, but universal, 30-point measure of cognitive status [12]. The MMSE has become one of the most widely used cognitive screening instruments for CD, which covers various cognitive domains. Specifically, the MMSE is used to estimate the severity of cognitive impairment and assess longitudinal changes in cognitive status. Trained neurologists or a neuropsychologist determined the MMSE scores as described previously [13]. A score ≤ 27 is considered reflective of cognitive impairment [14]. We were able to identify 34 participants as suitable for the CD group as they produced MMSE scores between 28–30 points in 2006–2008 and scores from 24–27 in 2012–2014. Similarly, 117 participants were defined as the control group, with scores from 28–30 in 2006–2008 that did not decrease when assessed in 2012–2014. The time between the two cognitive evaluations was not significantly different between the control (mean = 5.74 years) and CD (mean = 5.76 years) groups.

The verbal fluency test is a well-established method for evaluation of cognitive function [15]. All participants also completed a verbal fluency test. In this task, as in previous reports, the participants were asked to provide as many words beginning with Ta and Ka as they could recall [13].

### Medical information and blood biochemistry

The present study evaluated medical information obtained via self-administered questionnaires (education level, anamnesis at baseline and in 2012–2014, medication, frequency of depressive symptoms, smoking, and drinking habits). Instrumental activities of daily living (IADL) and metabolic equivalents (METs) were assessed as previously reported [16, 17]. The scoring guidelines recommend adding an additional point for people with less than 13 years of education [18]. Furthermore, blood chemistry data [triglycerides, total cholesterol, high-density lipoprotein cholesterol (HDL-C), non-HDL-C, total protein, albumin, A/G ratio, creatinine, uric acid, hemoglobin A1c (HbA1c), high sensitive C-reactive protein (hsCRP), and antibodies against *C. pneumonia*, and *H. pylori* antibodies] were assessed. An IgG index above 1.1, and an IgA index above 1.1, was defined as criteria for *C. pneumoniae* positivity [19], and a cutoff point greater than 2.3 for the ELISA VALUE indicated *H. pylori* positivity [20]. The following anthropometry data were also obtained during the health check-ups: weight, height, and systolic and diastolic blood pressure. Anamnesis and medication history were assessed using a questionnaire. Hypertension was resting systolic blood pressure ≥ 140 mmHg or being treated for hypertension. Diabetes mellitus was defined as HbA1c ≥ 6.5% and dyslipidemia as triglycerides ≥ 150 or HDL ≤ 40. Additionally, the pulse wave velocity [21], which is a potential marker of arterial stiffness, was measured in 2006–2008 and 2012–2014.

### Apolipoprotein E genotyping

Genomic DNA was extracted from the buffy coat fraction of each participant’s blood sample. Genotyping was performed using polymerase chain reaction (PCR) with the following primers; forward: ACGAGACCATGAAGGAGTTGAA and reverse: ACCTGCTCCTTCACCTCGTCCAG. Amplification of the genomic DNA resulted in a PCR product = 514 bp, which was subjected to a direct sequence or PCR-restriction fragment length polymorphism analysis [22]. The ApoE isoforms differed in cysteine and arginine content at positions 112 and 158: ApoE-ε2: Cys (TGC), Cys (TGC), ApoE-ε3: Cys (TGC), Arg (CGC), ApoE-ε4: Arg (CGC), Arg (CGC). ApoE status was classified as ε4 carriers for participants with the ApoE4 isoform (phenotypes ε2/4, ε3/4, ε4/4) and as non-4 carriers for participants without the ApoE4 isoform (phenotypes ε2/2, ε2/3, ε3/3).

### Scoring white matter and periventricular hyperintensities

Brain MRI was performed using a 1.5-T scanner. MRI was performed to assess different types of hyperintense signal abnormalities surrounding the ventricles, and deep white matter abnormalities were evaluated as deep white matter lesions (DWL) and periventricular hyperintensities (PVH), as previously reported [13]. MRI cerebrovascular staging was carried out using the Fazekas classification [23].

### Statistical analyses

Continuous variables are expressed as means ± standard deviations (SDs) or median [range], and categorical data are expressed as sums and percentages. Inter-group comparisons were performed using unpaired *t*-tests for continuous variables or Mann–Whitney U-tests, and the chi-square or Fisher’s exact tests for categorical variables (sex, ApoE4, education, depressive symptoms, baseline and 2012–2014 period anamnesis, *C. pneumonia* and *H. pylori* seropositivity, drinking and smoking prevalence, DWL, and PVH). Odds ratios (OR) and 95% confidence intervals (CI) were calculated using logistic regression analyses in which CD was the dependent variable and age, sex, ApoE4 status, education, smoking and drinking habits, and baseline anamnesis were the independent variables. Significant predictors from the logistic regression analysis were considered independent variables in the multiple logistic regression analysis using a stepwise forward selection method. A Spearman’s rank correlation coefficient was calculated to confirm whether serum A/G ratio was related to MMSE scores, pulse wave velocity, and hsCRP, as well as significant variables from the logistic regression analysis. All statistical tests were two-tailed, and differences with a *p*-value < 0.05 were considered statistically significant. SPSS software (version 18.0) was used for all statistical analyses.

## Results

### Participant characteristics

Table 1 shows participant characteristics, including anthropometric measures, blood chemistry data, questionnaire responses, and the number for each item between the control and CD groups. The mean age (± standard deviation: SD) for the control group was 59.4 (±5.9) years, compared to 61.2 (±4.6) years for the CD group. There were no significant differences between the anthropometric measures of the two groups. Although the CD group did not show significantly decreased scores on verbal fluency tasks in 2006–2008, their verbal fluency scores significantly decreased in 2012–2014. Furthermore, no significant differences in depressive symptoms, IADL, or METs were observed between the control and CD groups. The distribution of ApoE4 genotypes was in the Hardy–Weinberg equilibrium (control group: *p* = 0.621; CD group: *p* = 0.565). The ApoE4 allele distribution was not significantly different between the control and CD groups.

**Table 1:** Participant characteristics at baseline and follow-up according to CD condition

	All					Male					Female				
	Number		Data are mean ± SD, median [range] or (%)		*p* value	Number		Data are mean ± SD, median [range] or (%)		*p* value	Number		Data are mean ± SD, median [range] or (%)		*p* value
	Control	CD	Control	CD		Control	CD	Control	CD		Control	CD	Control	CD	
Baseline															
Age (years)	117	34	59.4 ± 5.9	61.2 ± 4.6	0.066	82	19	59.9 ± 6.2	61.2 ± 4.6	0.128	35	15	58.2 ± 5.3	59.9 ± 4.3	0.280
Sex (Female)	35	15	29.9 (%)	44.1 (%)	0.148										
BMI (kg/m^2^)	117	34	22.3 ± 2.4	22.6 ± 3.3	0.590	82	19	22.5 ± 2.3	23.0 ± 2.1	0.379	35	15	21.8 ± 2.8	22.0 ± 4.4	0.844
SBP (mmHg)	117	34	124 ± 18.6	124 ± 19.0	0.974	82	19	124 ± 15.7	130 ± 20.8	0.118	35	15	124 ± 24.4	116 ± 12.9	0.122
DBP (mmHg)	117	34	72.8 ± 9.5	73.0 ± 10.9	0.916	82	19	73.8 ± 8.9	74.6. ± 12.5	0.804	35	15	70.4 ± 10.6	71.0 ± 8.43	0.852
Triglyceride (mg/dl)	115	34	98.7 ± 52.3	103 ± 47.4	0.604	81	19	102 ± 56.2	120 ± 51.8	0.198	34	15	90.1 ± 40.8	82.7 ± 31.5	0.537
Total cholesterol (mg/dl)	115	34	211 ± 30.1	224 ± 43.2	0.108	81	19	208 ± 28.8	209 ± 37.4	0.938	34	15	219 ± 32.2	244 ± 42.9	**0.029**
HDL-C (mg/dl)	115	34	68.0 ± 18.4	63.5 ± 16.0	0.205	81	19	64.1 ± 18.4	55.7 ± 11.5	0.061	34	15	77.1 ± 15.2	73.4 ± 15.6	0.437
non-HDL-C (mg/dl)	115	34	141 ± 37.7	161 ± 42.4	**0.009**	82	19	142 ± 36.5	153 ± 40.5	0.259	35	15	138 ± 40.8	171 ± 43.9	**0.014**
Total Protein (g/dl)	115	34	7.14 ± 0.39	7.33 ± 0.33	**0.012**	81	19	7.11 ± 0.39	7.22 ± 0.31	0.276	34	15	7.22 ± 0.37	7.47 ± 0.32	**0.027**
Albumin (g/dl)	115	34	4.40 ± 0.21	4.41 ± 0.19	0.751	81	19	4.42 ± 0.22	4.39 ± 0.23	0.626	34	15	4.36 ± 0.18	4.45 ± 0.15	0.137
A/G ratio	99	30	1.87 ± 0.21	1.74 ± 0.16	**0.002**	71	18	1.91 ± 0.22	1.78 ± 0.16	**0.025**	28	12	1.78 ± 0.17	1.68 ± 0.16	0.084
Creatinine (mg/dl)	115	34	0.94 ± 0.14	0.94 ± 0.23	0.850	81	19	1.00 ± 0.12	1.04 ± 0.25	0.277	34	15	0.81 ± 0.10	0.80 ± 0.11	0.816
Uric acid (mg/dl)	115	34	5.47 ± 1.18	5.30 ± 1.37	0.466	81	19	5.89 ± 1.03	5.75 ± 1.54	0.620	34	15	4.47 ± 0.91	4.73 ± 0.87	0.369
HbA1c	117	34	5.05 ± 0.77	5.34 ± 0.66	**0.044**	82	19	5.09 ± 0.71	5.43 ± 0.81	0.076	35	15	4.98 ± 0.88	5.24 ± 0.41	0.228
hsCRP (mg/dl)	71	26	0.09 ± 0.07	0.11 ± 0.11	0.347	54	15	0.08 ± 0.07	0.13 ± 0.13	0.183	17	11	0.10 ± 0.08	0.08 ± 0.09	0.559
*C. pneumoniae* seropositivity	39	14	33.3 (%)	41.2 (%)	0.420	25	11	30.5 (%)	57.9 (%)	**0.034**	14	3	40.0 (%)	20.0 (%)	0.209
*H. pylori* seropositivity	58	27	49.6 (%)	79.4 (%)	**0.003**	37	15	45.1 (%)	78.9 (%)	**0.010**	21	12	60.0 (%)	80.0 (%)	0.209
ApoE4 carrier	25	6	21.4 (%)	17.7 (%)	0.633	15	4	18.2 (%)	21.1 (%)	0.756	10	2	28.6 (%)	13.3 (%)	0.466
ApoE4 not determined	2	1	1.71 (%)	2.94 (%)		2	0	2.43 (%)	0 (%)		0	1	0 (%)	6.67 (%)	
Anamnesis															
Hypertension	35	14	29.9 (%)	41.2 (%)	0.298	22	11	26.8 (%)	57.9 (%)	**0.015**	13	3	37.1 (%)	20.0 (%)	0.328
Hyperlipidemia	18	6	15.3 (%)	17.6 (%)	0.793	15	5	18.3 (%)	26.3 (%)	0.525	3	1	8.57 (%)	6.67 (%)	1.000
Diabetes	21	8	18.0 (%)	23.5 (%)	0.471	17	5	20.7 (%)	26.3 (%)	0.759	4	3	11.4 (%)	20.0 (%)	0.415
History of stroke	1	0	0.86 (%)	0 (%)	1.000	0	0	0 (%)	0 (%)		1	0	2.86 (%)	0 (%)	1.000
Education															
< 13 year	53	23	45.3 (%)	67.6 (%)	**0.049**	32	12	39.0 (%)	63.2 (%)	0.122	21	11	60.0 (%)	73.3 (%)	0.526
≥ 13 year	60	11	51.3 (%)	32.4 (%)		47	7	57.3 (%)	36.8 (%)		13	4	37.1 (%)	26.7 (%)	
Not determined	4	0	3.42 (%)	0 (%)		3	0	3.66 (%)	0 (%)		1	0	2.86 (%)	0 (%)	
Alcohol drinking															
Current	78	15	66.7 (%)	44.1 (%)	0.067	67	13	81.7 (%)	68.4 (%)	0.301	11	2	31.4 (%)	13.3 (%)	0.297
Former	3	2	2.56 (%)	5.88 (%)		3	2	3.66 (%)	10.5 (%)		0	0	0 (%)	0 (%)	
Never	35	16	29.9 (%)	47.0 (%)		11	4	13.4 (%)	21.1 (%)		24	12	68.6 (%)	80.0 (%)	
Not determined	1	1	0.86 (%)	2.94 (%)		1	0	1.22 (%)	0 (%)		0	1	0 (%)	6.67 (%)	
Smoking (%)															
Current	21	7	17.9 (%)	20.6 (%)	0.758	20	7	24.4 (%)	36.8 (%)	0.561	1	0	2.86 (%)	0 (%)	0.221
Former	40	9	34.2 (%)	26.5 (%)		40	8	48.8 (%)	42.1 (%)		0	1	0 (%)	6.67 (%)	
Never	54	16	46.2 (%)	47.1 (%)		21	4	25.6 (%)	21.1 (%)		33	12	94.3 (%)	80.0 (%)	
Not determined	2	2	1.71 (%)	5.88 (%)		1	0	1.22 (%)	0 (%)		1	2	2.86 (%)	13.3 (%)	
In 2006-2008															
MMSE	117	34	29.5 ± 0.71	29.0 ± 0.75	**<0.001**	82	19	29.6 ± 0.64	29.3 ± 0.67	0.092	35	15	29.4 ± 0.85	28.6 ± 0.72	**0.004**
Ta	117	34	10 [4-18]	9 [4-18]	0.274	82	19	10 [4-18]	9 [4-18]	0.501	35	15	10 [4-18]	8 [4-18]	0.469
Ka	117	34	12 [2-21]	11 [5-17]	0.052	82	19	12 [4-21]	11 [5-17]	0.100	35	15	12 [2-21]	10 [6-17]	0.425
Pulse wave velocity (m/sec)	116	34	15.2 ± 2.68	16.6 ± 2.73	**0.013**	81	19	15.4 ± 2.56	17.8 ± 2.94	**<0.001**	35	15	14.9 ± 2.95	14.9 ± 1.25	0.959
In 2012-2014															
MMSE	117	34	29.5 ± 0.67	26.0 ± 0.88	**<0.001**	82	19	29.6 ± 0.54	25.8 ± 0.87	**<0.001**	35	15	29.3 ± 0.87	26.1 ± 0.91	**<0.001**
Verbal fluency tasks															
Ta	117	34	9 [2-17]	8 [3-24]	**0.026**	82	19	9 [2-15]	8 [4-13]	0.055	35	15	9 [2-17]	8 [3-24]	0.237
Ka	117	34	10 [3-20]	9 [5-18]	**0.038**	82	19	10 [3-20]	9 [5-18]	0.440	35	15	10 [3-20]	8 [5-14]	**0.017**
Pulse wave velocity (m/sec)	116	34	17.0 ± 2.99	18.5 ± 3.28	**0.011**	81	19	17.2 ± 2.65	19.4 ± 3.76	**0.026**	35	15	16.4 ± 3.64	17.4 ± 2.20	0.329
IADL	117	34	13 [9-13]	13 [7-13]	0.463	81	19	13 [9-13]	13 [9-13]	0.595	35	15	13 [10-13]	13 [7-13]	0.580
METs	117	34	14.5 [2.0-48.4]	16.9 [1.5-50.4]	0.161	81	19	15.2 [2.5-48.4]	17.1 [1.5-50.4]	0.195	35	15	14.5 [2.0-42.5]	14.2 [1.5-40.0]	0.335
Anamnesis															
Hypertension	63	20	53.9 (%)	58.8 (%)	0.697	45	15	54.9 (%)	78.9 (%)	0.071	18	5	51.4 (%)	33.3 (%)	0.355
Hyperlipidemia	61	25	52.1 (%)	73.5 (%)	**0.031**	41	14	50.0 (%)	73.7 (%)	0.076	20	11	57.1 (%)	73.3 (%)	0.351
Diabetes	26	10	22.2 (%)	29.4 (%)	0.372	22	7	26.8 (%)	36.8 (%)	0.407	4	3	11.4 (%)	20.0 (%)	0.415
History of stroke	6	1	5.13 (%)	2.94 (%)	0.822	4	1	4.88 (%)	5.26 (%)	1.000	2	0	5.71 (%)	0 (%)	0.640
Feel depression															
Nothing	108	31	92.3 (%)	91.2 (%)	0.804	77	18	93.9 (%)	94.7 (%)	0.888	31	13	88.6 (%)	86.7 (%)	1.000
Sometimes	8	3	6.84 (%)	8.82 (%)		4	1	4.88 (%)	5.26 (%)		4	2	11.4 (%)	13.3 (%)	
Always	1	0	0.86 (%)	0 (%)		1	0	1.22 (%)	0 (%)		0	0	0 (%)	0 (%)	

Category differences are analyzed by *t*-test, IADL, METs and Verbal fluency tasks are analyzed by U-test

Chi-square test for ApoE, smoking and alcohol drinking habit, or Fisher’s exact test for sex, *H. pylori* seropositivity, *C. pneumoniae* seropositivity, hypertension, hyperlipidemia, diabetes, history of stroke, education

*CD* cognitive decline, *MMSE* Mini-Mental State Examination, *A/G* albumin to globulin, *H. pylori Helicobacter pylori*, *C. pneumoniae Chlamydia pneumoniae*, *ApoE* apolipoprotein E, *SD* standard deviation, *BMI* body mass index, *SBP* systolic blood pressure, *DBP* diastolic blood pressure, *IADL* instrumental activities of daily living, *METs* metabolic equivalents, *HDL-C* high-density lipoprotein cholesterol, *HbA1c* hemoglobin A1c, *hsCRP* high sensitive C-reactive protein

### Associations between CD and control participant characteristics

Non-HDL-C, total protein, HbA1c, *H. pylori* seropositivity, and pulse wave velocity during both 2006–2008 and 2012–2014 were significantly higher in the CD group compared to the control group (Table 1). In contrast, the A/G ratio was significantly lower in the CD group (Table 1).

To determine variables significantly associated with CD, a logistic regression analysis adjusted for age, sex, ApoE4 status, education, smoking and alcohol drinking habits, and anamnesis was performed. The variables selected by this analysis were MRI evaluation, body mass index (BMI), systolic blood pressure (SBP), diastolic blood pressure (DBP), triglycerides, total cholesterol, HDL, non-HDL, total protein, albumin, A/G ratio, creatinine, uric acid, HbA1c, hsCRP, *C. pneumoniae* and *H. pylori* seropositivity, pulse wave velocity, education, and ApoE4 status (Tables 2 and 3). From a diagnostic imaging viewpoint (Table 2), the odds of DWL grade 1 and 2, which were evaluated by a Fazekas classification during the 2nd follow-up, showed significant higher values for CD group. As shown in Table 3, non-HDL-C, A/G ratio, HbA1c, and *H. pylori* seropositivity were predictive of CD.

**Table 2:** Logistic regression analysis according to CD condition

	Number		Model I		Model II		Model III	
	Control	CD	OR	95% CI	OR	95% CI	OR	95% CI
DWL Baseline								
grade 0	67	17	Reference		Reference		Reference	
grade 1	44	17	1.476	0.669-3.255	1.684	0.715-3.967	1.451	0.573-3.671
DWL 1st follow-up								
grade 0	52	11	Reference		Reference		Reference	
grade 1	55	20	1.764	0.754-4.129	1.872	0.759-4.619	1.763	0.697-4.455
grade 2	8	3	1.314	0.620-2.787	1.458	0.598-3.557	1.049	0.299-3.387
DWL 2nd follow-up								
grade 0	43	5	Reference		Reference		Reference	
grade 1	50	21	**3.659**	**1.242-10.77**	**4.562**	**1.382-15.05**	**4.427**	**1.323-14.81**
grade 2	17	7	**2.058**	**1.042-4.062**	**3.969**	**1.424-11.06**	**4.215**	**1.384-12.83**
grade 3	6	1	1.008	0.450-2.259	0.807	0.273-2.384	1.103	0.274-4.442
PVH Baseline								
grade 0	83	23	Reference		Reference		Reference	
grade 1	29	11	1.156	0.479-2.791	0.848	0.327-2.197	0.700	0.254-1.930
PVH 1st follow-up								
grade 0	66	18	Reference		Reference		Reference	
grade 1	41	15	1.152	0.498-2.668	0.971	0.392-2.409	0.909	0.359-2.298
grade 2	8	1	0.591	0.192-1.815	0.611	0.188-1.988	0.632	0.191-2.095
PVH 2st follow-up								
grade 0	61	17	Reference		Reference		Reference	
grade 1	44	13	0.857	0.354-2.075	0.847	0.337-2.131	0.857	0.337-2.175
grade 2	7	4	1.450	0.716-2.937	1.254	0.554-2.842	1.221	0.524-2.841

ModelI: Adjusted for age and sex

Model II: Adjusted for age, sex, apolipoprotein E4, education, smoking and alcohol drinking habits

Model III: Adjusted for age, sex, apolipoprotein E4, education, smoking and alcohol drinking habits, hypertension, hyperlipidemia and diabetes

*CD* cognitive decline, *DWL* white matter lesions, *PVH* perivascular hyperintensities, *OR* odds ratio, *CI* confidence interval

**Table 3:** Logistic regression analysis according to CD condition

	Number		Model I		Model II		Model III	
	Control	CD	OR	95% CI	OR	95% CI	OR	95% CI
BMI	117	34	1.073	0.998-1.154	1.060	0.910-1.235	1.024	0.868-1.207
SBP	117	34	0.999	0.938-1.151	0.994	0.971-1.017	0.971	0.941-1.002
DBP	117	34	1.010	0.970-1.052	0.998	0.954-1.044	0.997	0.941-1.036
Triglyceride	115	34	1.003	0.996-1.011	1.002	0.994-1.010	1.002	0.989-1.015
Total cholesterol	115	34	1.001	0.998-1.022	1.009	0.995-1.022	1.009	0.995-1.022
HDL-C	115	34	**0.972**	**0.946-0.999**	0.977	0.950-1.004	0.973	0.942-1.005
non-HDL-C	115	34	**1.014**	**1.003-1.025**	**1.013**	**1.001-1.025**	**1.013**	**1.001-1.027**
Total Protein	115	34	**2.971**	**1.023-8.622**	**3.575**	**1.088-11.74**	3.219	0.938-11.04
Albumin	115	34	2.035	0.291-14.21	1.980	0.243-16.12	1.852	0.218-15.71
A/G ratio	99	30	**0.063**	**0.006-0.619**	**0.032**	**0.003-0.379**	**0.037**	**0.003-0.470**
Creatinine	115	34	2.688	0.207-34.86	2.852	0.185-43.87	2.235	0.135-36.88
Uric acid	115	34	1.007	0.695-1.459	1.037	0.707-1.520	1.068	0.730-1.563
HbA1c	117	34	**2.433**	**1.156-5.118**	**2.405**	**1.131-5.112**	**2.586**	**1.036-6.455**
hsCRP	71	26	12.95	0.104-1617	66.97	0.303-14824	42.42	0.127-14225
*C. pneumoniae* seropositivity	117	34	1.297	0.580-2.899	1.437	0.619-3.336	1.593	0.664-3.82
*H. pylori* seropositive	117	34	**3.507**	**1.398-8.801**	**4.867**	**1.754-13.50**	**4.786**	**1.710-13.39**
Pulse wave velocity in 2006-2008	116	34	**1.184**	**1.021-1.371**	**1.209**	**1.028-1.422**	1.179	0.989-1.404
Pulse wave velocity in 2012-2014	116	34	**1.158**	**1.015-1.322**	1.145	0.989-1.327	1.125	0.963-1.313
Education	113	34	2.129	0.927-4.890				
ApoE4 carrier	115	33	0.781	0.310-1.970				

Model I: Adjusted for age and sex

Model II: Adjusted for age, sex, apolipoprotein E4, education, smoking and alcohol drinking habits

Model III: Adjusted for age, sex, apolipoprotein E4, education, smoking and alcohol drinking habits, hypertension, hyperlipidemia and diabetes

*CD* cognitive decline, *OR* odds ratio, *CI* confidence interval, *A/G* albumin to globulin, *H. pylori Helicobacter pylori*, *C. pneumoniae Chlamydia pneumoniae*, *ApoE* apolipoprotein E, *BMI* body mass index, *SBP* systolic blood pressure, *DBP* diastolic blood pressure, *HDL-C* high-density lipoprotein cholesterol, *HbA1c* hemoglobin A1c, *hsCRP* high sensitive C-reactive protein

Next, a multivariate analysis was performed with all of the significant variables considered simultaneously: non-HDL-C, A/G ratio, HbA1c, and *H. pylori* seropositivity (Table 4). Based on a stepwise forward selection method, A/G ratio was significantly predictive with a low OR (OR = 0.092, 95% CI = 0.010–0.887), and *H. pylori* seropositivity was significantly predictive with a high OR (OR = 4.468, 95% CI = 1.535–13.00). Therefore, A/G ratios were significantly positive correlation of MMSE scores (during both 2006–2008 and 2012–2014), and negative correlation with non-HDL-C, HbA1c, and hsCRP (Table 5).

**Table 4:** Multiple logistic regression analysis with stepwise forward selection based on CD condition

	Multivariate		Stepwise forward selection	
	OR	95% CI	OR	95% CI
non-HDL-C	1.011	0.999-1.024		
A/G ratio	0.265	0.022-3.215	**0.092**	**0.010-0.887**
HbA1c	1.743	0.782-3.883		
*H. pylori* seropositive	**4.255**	**1.422-12.73**	**4.468**	**1.535-13.00**
Sex	1.493	0.522-4.270		
Age	1.079	0.983-1.183		

*CD* cognitive decline, *OR* odds ratio, *CI* confidence interval, *HDL-C* high-density lipoprotein cholesterol, *HbA1c* hemoglobin A1c, *A/G* albumin to globulin, *H. pylori*: *Helicobacter pylori*

**Table 5:** Correlations between A/G ratio and variables used in the multivariate analysis

	A/G ratio	
	Coefficient	*p* value
MMSE score in 2006-2008	0.187	**0.034**
MMSE score in 2012-2014	0.264	**0.003**
non-HDL-C	−0.230	**0.009**
HbA1c	−0.193	**0.029**
hsCRP	−0.369	**0.001**
Pulse wave velocity in 2006-2008	−0.047	0.598
Pulse wave velocity in 2012-2014	−0.001	0.989

*A/G* albumin to globulin, *MMSE* Mini-Mental State Examination, *HDL-C* high-density lipoprotein cholesterol, *HbA1c* hemoglobin A1c, *hsCRP* high sensitive C-reactive protein

## Discussion

Although no single cause for cognitive impairment has been identified, recent research suggests that several pathogenetic factors such as aging, genetics, inflammation, dyslipidemia, diabetes, and infectious diseases are plausible candidates. The present results revealed that *H. pylori* seropositivity tended to be related to more severe CD incidence. Furthermore, the present study explored, for the first time, an association between A/G ratios and CD incidence.

Growing evidence has underscored a mechanistic link between cholesterol metabolism in the brain and the formation of amyloid plaques. Excess brain cholesterol has been associated with increased formation and deposition of β-amyloid from amyloid precursor proteins. Indeed, non-HDL-C was associated with CD incidence in the present study. Cholesterol-lowering statins have become a focus for AD research [24]. Moreover, genetic polymorphisms associated with pivotal points in cholesterol metabolism within brain tissues may contribute to AD risk and pathogenesis. A recent meta-analysis indicated the positive predictive value of the ApoE4 allele for progression from cognitive impairment to AD-type dementia [25]. Although there is convincing evidence to suggest that ApoE4 is the main predictor for progression from CD to AD, ApoE4 may not be a risk factor for CD incidence. For instance, the present findings revealed that ApoE4 status was not associated with CD incidence.

Cognitive impairment can present with mild deficits affecting one or multiple cognitive domains. Size and location of white matter lesions and ischemic and hemorrhagic strokes are associated with varying clinical presentation in these patients [26]. Concerning the link between CD incidence and cerebrovascular lesion occurrence, we found that the CD group showed not only decreased MMSE scores but also progression of DWL Fazekas grade. In general, white matter lesions are a key vascular, cognitive impairment marker. Although DWL and PVH were not predictive of CD incidence in the present study, CD group indicated DWL grade progression.

Recent studies have shown that *H. pylori* infection leads to cognitive impairment [3]. *H. pylori* infection likely influences cognitive impairment by increasing neurodegenerative lesions, especially neurofibrillary tangles and neuronal loss via ischemic lesions. *H. pylori* infection evolving over many years could also cause chronic gastric and plasmatic inflammation, thus inducing a chronic inflammation model plausibly responsible for cerebrovascular lesions and the exacerbation of neurodegeneration [3]. Moreover, when accomplished, *H. pylori* eradication is beneficial for improving cognitive and functional states among patients, perhaps altering the progressive nature of AD [27]. Additionally, chronic inflammation might be an underlying factor for an association between metabolic syndrome and CD [28]. The present study suggests a relationship between inflammation, disruption of homeostatic factors [e.g., cholesterol metabolism (dyslipidemia), HbA1c (diabetes), and *H. pylori* seropositivity (infectious disease)] and cognitive function, since these inflammatory mechanisms are also hypothesized to be involved in the pathogenesis of cognitive impairment. Furthermore, inflammation may also promote the development and progression of atherosclerotic plaques [8], which is in line with evidence suggesting a link between cognitive impairment and atherosclerosis [9]. However, in the present study, pulse wave velocity in 2006–2008 was not predictive of CD. In other words, disruption of homeostatic factors, in itself, was a more useful predictor of CD incidence than arterial stiffness.

From a preventive viewpoint, albumin serves as an antioxidant, eliminates toxins, and inhibits the formation of amyloid beta-peptide fibrils. Several studies suggest that low albumin levels are associated with a risk for cognitive impairment and dementia [29, 30]. The present study, however, observed that CD incidence was associated with A/G ratios but not albumin. In fact, albumin levels did not differ between the control and CD groups. Additionally, total protein levels trended toward a risk for CD incidence, indicating that globulin levels were increased in the CD group due to no difference in albumin levels between the control and CD groups. Namely, A/G ratios may decrease due to globulin levels rising during chronic inflammation. Similarly, increased serum globulins have been associated with cancer, rheumatoid diseases, chronic liver disease, nephrotic syndrome, and diabetes mellitus; decreased albumin has been associated with chronic infections, chronic liver disease, and nephrotic syndrome [31, 32]. Thus, it appears that the modification of albumin and globulin is associated with disruption of homeostasis. In the present study, A/G ratios were also significantly and positively correlated with MMSE scores and negatively correlated with cholesterol metabolism, HbA1c, and hsCRP. These factors were decreased in relation to CD incidence based on our stepwise regression analysis. In sum, the A/G ratio may be a very reliable index for CD incidence caused by disruption of homeostasis.

A few study limitations should be noted. First, there were a relatively small number of participants in the CD group. Therefore, an analysis of data from male and female participants separately would not be useful because of the low statistical power. Although, the proportion of male and female participants, and the education level of the participants differed between the two groups, logistic regression analysis was performed after adjusting for these variables. While a study with low statistical power has a reduced likelihood of detecting a true effect, nested case–control studies with small sample sizes are still widely conducted and can be used to identify candidate targets. Secondly, we diagnosed *H. pylori* infections via serum antibody detection, whereas the gold standard involves gastric testing. The primary limitation of this serologic test is its inability to discriminate between current and old infections. However, *H. pylori* induces humoral and cellular immune responses that can affect or perpetuate neural tissue damage [33]. This pathogen may influence the pathophysiology of AD by inducing vascular disorders that have been implicated in endothelial damage and neurodegeneration. Overall, the results of the present and previous studies suggest that both current and old *H. pylori* infections contribute to CD by inducing neural tissue damage. One other issue was that A/G ratios, as well as other biological markers, were only determined once, during the baseline survey. Conversely, cognitive data were available at both baseline and follow-up. Therefore, larger prospective trials are needed to better assess how A/G ratios are associated with CD incidence.

## Conclusions

The current study observed that A/G ratios, which are part of routinely administered laboratory tests, could reflect changes in homeostatic factors. Additional investigations are expected to show that the modification of A/G ratios could lead toward novel and effective strategies for predictive CD screening.

## Abbreviations

A/G: Albumin to globulin
AD: Alzheimer’s disease
ApoE: Apolipoprotein E
BMI: Body mass index
*C. pneumoniae*: *Chlamydia pneumoniae*
CD: Cognitive decline
CI: Confidence interval
DBP: Diastolic blood pressure
DWL: White matter lesions
*H. pylori*: *Helicobacter pylori*
HbA1c: Hemoglobin A1c
HDL-C: High-density lipoprotein cholesterol
hsCRP: High sensitive C-reactive protein
IADL: Instrumental activities of daily living
METs: Metabolic equivalents
MMSE: Mini-mental state examination
MRI: Magnetic resonance imaging
OR: Odds ratio
PVH: Periventricular hyperintensities
SBP: Systolic blood pressure
SD: Standard deviation
